# Electronic-Nose Technology for Lung Cancer Detection: A Non-Invasive Diagnostic Revolution

**DOI:** 10.1007/s00408-025-00828-0

**Published:** 2025-07-08

**Authors:** A. M. Dhanush Gowda, Akanksha D. Dessai, Usha Y. Nayak

**Affiliations:** Department of Pharmaceutics, Manipal College of Pharmaceutical Sciences, Manipal Academy of Higher Education, Manipal, 576104 Karnataka India

**Keywords:** E-nose, Lung cancer, Volatile organic compounds, Non-invasive, Lung cancer diagnosis

## Abstract

**Background:**

Lung cancer (LC) remains a leading cause of cancer-related mortality worldwide, primarily due to late-stage diagnosis and the absence of effective early detection methods.

**Objective:**

This review aims to explore the principles, technological advancements, current limitations, and future prospects of electronic nose (E-nose) systems in the early detection of lung cancer.

**Methods:**

The review analyzes recent literature on E-nose devices that detect volatile organic compounds (VOCs) in exhaled breath, focusing on their integration with artificial intelligence (AI) and machine learning for pattern recognition and diagnostic classification.

**Results:**

E-noses have demonstrated high sensitivity and specificity in differentiating cancerous from non-cancerous breath samples. However, challenges such as sensor stability, lack of standardization in breath collection, demographic variability, and the need for large training datasets for AI models limit their clinical adoption.

**Conclusion:**

Despite current limitations, E-nose technology shows strong potential as a rapid, non-invasive, and cost-effective tool for early LC screening. Enhancing sensor durability, improving data processing, and conducting large-scale validation studies are critical next steps. Integration with imaging and molecular biomarkers may further improve diagnostic accuracy and clinical utility.

**Graphical Abstract:**

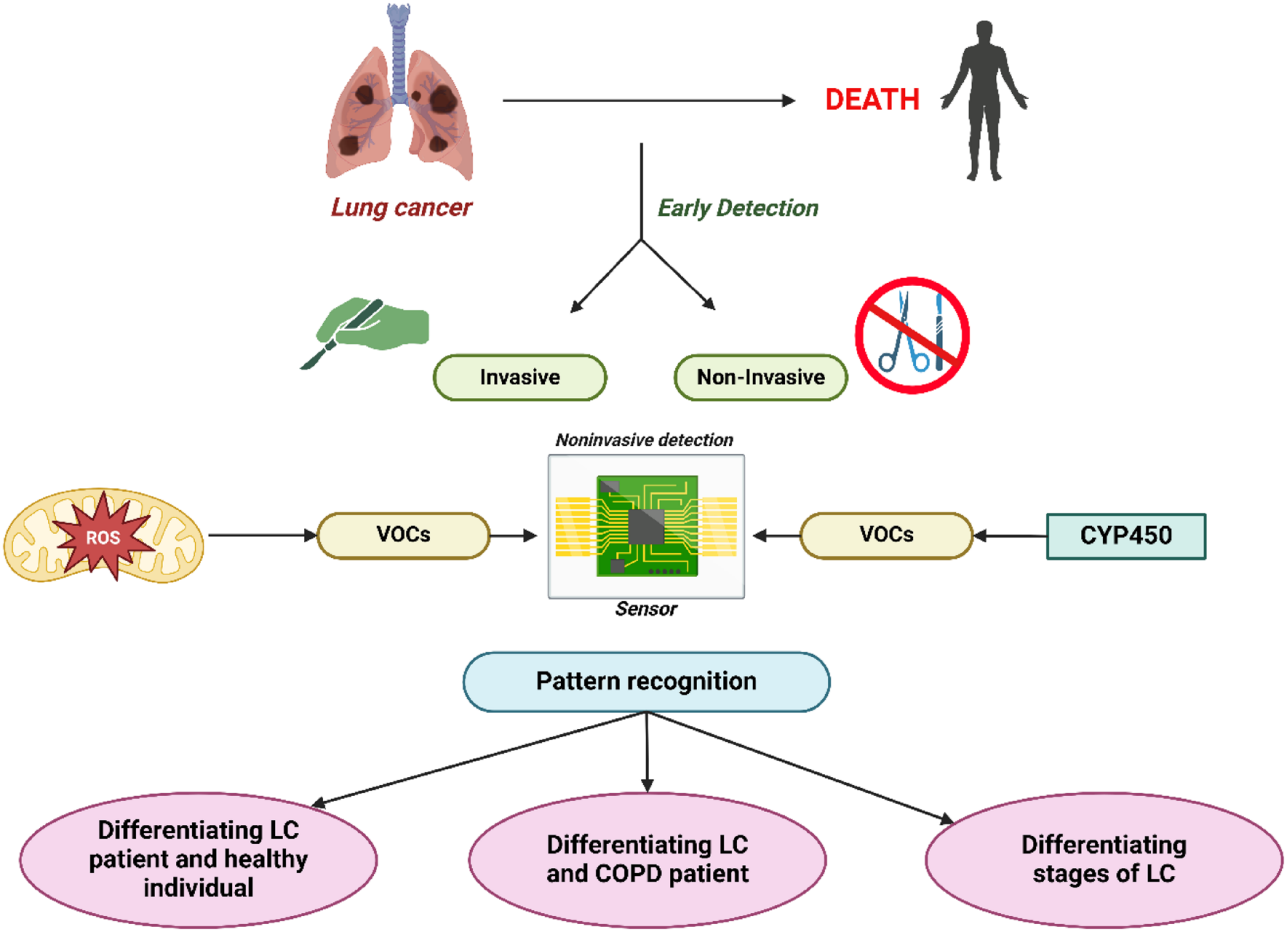

## Introduction to Electronic Nose

Lung cancer (LC) is an aggressive and multifaceted disease and affects every patient differently. The disease grows quickly and can sometimes be challenging to treat, meaning that no case is alike from the next one [1]. According to GLOBOCAN 2020, there were approximately 2.2 million new cases of LC (11.4%) and nearly 1.8 million LC-related deaths (18.0%) in 2020 [2]. LC was the second most prevalent cancer diagnosis globally in 2020, accounting for 2,206,771 new cases annually, (11.6% of all cancer cases), according to the Global Cancer Observatory, yet responsible for 1,796,144 fatalities (18% of all cancer-related deaths), making it the top cause of cancer-related mortality [3]. 72,510 instances of cancer occur annually in India, accounting for 5.8% of all cancer cases [4].

**Fig. 1:**
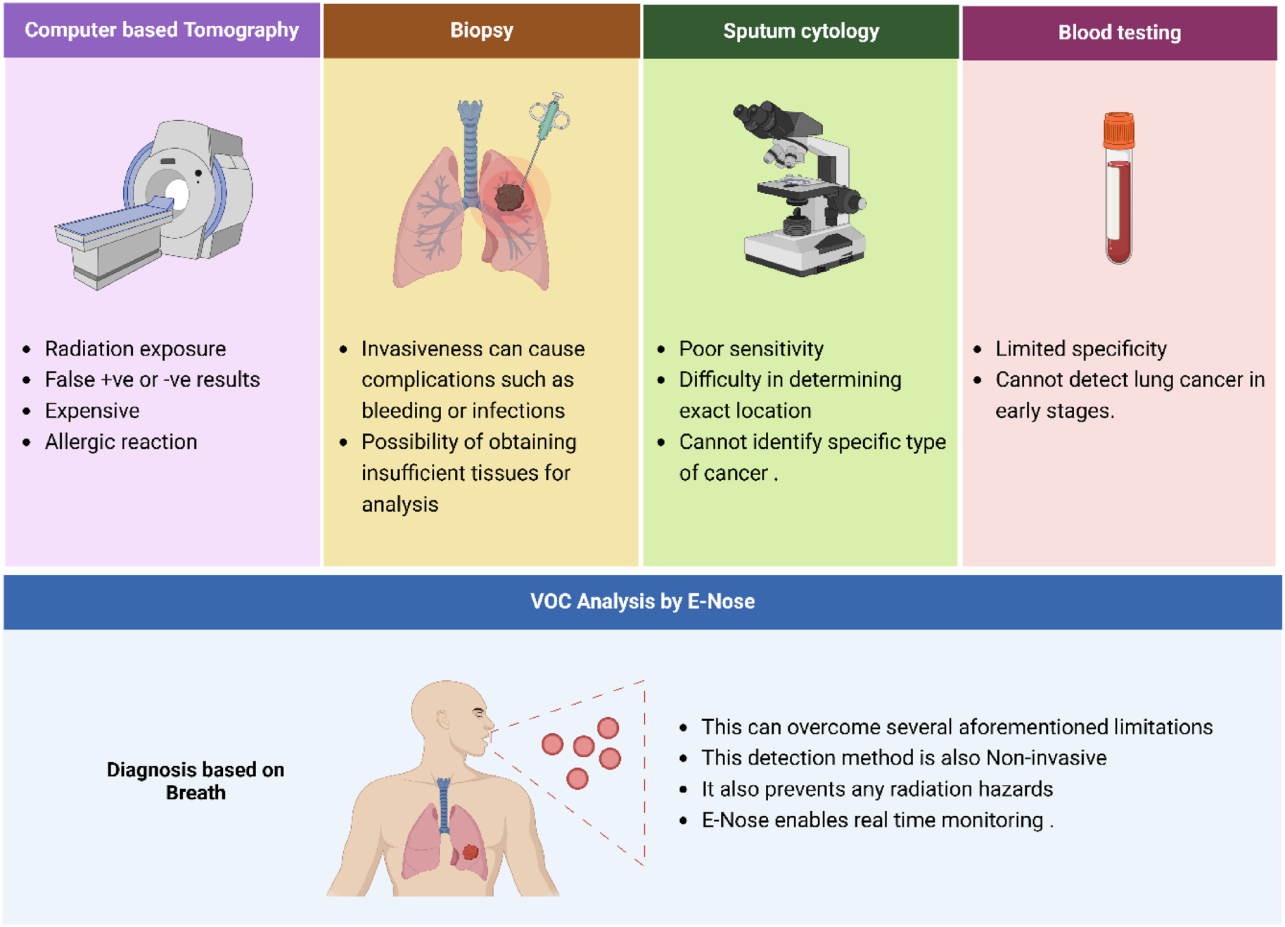
Evolution of Lung Cancer Diagnostic Techniques: From Traditional to Modern Approaches

LC can be treated with surgery, radiation therapy, chemotherapy, stereotactic body radiotherapy, targeted therapy, and immunotherapy [5, 6]. Despite the availability of these highly advanced treatments, the annual mortality rate from LC in India is 66,279 [4]. The early detection of LC and other forms of cancer plays a vital role in improving patient survival rates and reducing the associated socio-economic burdens, such as high medical costs and loss of productivity. In the case of stage 1 LC, the likelihood of a cure can be as high as 70%. Therefore, the development of advanced diagnostic methods to identify LC at an early stage will be instrumental in enhancing future screening programs, ultimately leading to better patient outcomes and a reduced healthcare burden [3, 4]. Computer-based tomography (CT), biopsy, bronchoscopy, blood testing, and sputum cytology are some of the diagnostic techniques used to identify LC [7]. However, each of these techniques has drawbacks, including the potential for misinterpretation, being time-consuming, costly, and causing discomfort for patients, which are illustrated in Fig. 1 [8, 9]. Therefore, researchers are actively exploring innovative methods for detecting LC, as the currently available techniques are invasive and often uncomfortable for patients [10]. In this regard, an E-nose presents a promising non-invasive alternative, addressing these limitations while potentially improving early diagnosis [11].

The detection of odors through technological devices is often referred to as "Electronic-nose” or “E-nose” [12]. An E-nose was initially introduced by Dodd and Persaud as a mechanism to replicate the capacity of the animal olfactory system to differentiate between various scents [13]. Despite significant differences in working principle, sensor count, sensitivity, and selectivity compared to the mammalian nose, it employs sensors to detect gases and transmits these signals to a recognition organ, such as the brain or a computer [14, 15]. The E-nose identifies fingerprints, or breath prints, through the activation of gas sensors that respond to volatile organic compounds (VOC). These compounds serve as indicators of biological metabolism and can be present in various organic fluids and exhaled breath [16].

The objective of this review is to explore innovative, non-invasive, and patient-friendly approaches for LC detection and management. Additionally, it aims to examine the applications of E-nose technology, discuss ongoing clinical trials, and highlight its current implementations. Furthermore, this review will address the challenges associated with E-nose technology, propose potential solutions, and explore future directions for its advancement in medical diagnostics.

We conducted a comprehensive literature search using electronic databases such as Google Scholar, PubMed, and Scopus. The search spanned publications until 2025, with particular emphasis on studies retrieved from Scopus. The search terms included combinations of keywords such as *“electronic nose,” “E-nose,” “lung cancer detection,” “volatile organic compounds (VOCs),” “non-invasive diagnostics,” “E-nose sensors,”* and *“machine learning in breath analysis.”* Studies were selected based on their relevance to the development, application, clinical evaluation, and technological advancement of E-nose systems for lung cancer diagnosis. We included peer-reviewed research articles, including original studies, reviews, and clinical trial reports, that focused on the use of E-nose technology for lung cancer detection. Exclusion criteria involved non-English publications, non-human studies, conference abstracts without full texts, and articles not directly related to E-nose applications in lung cancer.

The reviewed studies provided the foundation for this article, which outlines the working principles, sensor technologies, advantages, clinical relevance, and future potential of E-nose systems in lung cancer detection.

## Technology behind E-noses for LC detection

Breath analysis is an emerging field, with most studies conducted in recent years. It has long been recognized that human breath contains hundreds of VOCs [17]. In the 1980s, early research established a link between elevated VOC levels and LC, fuelling interest in identifying specific cancer biomarkers [18–20]. While traditional techniques like gas chromatography-mass spectrometry (GC–MS) are highly effective, they are also expensive and complex [21]. This has prompted researchers to explore alternative approaches, such as nanomaterial-based sensor arrays, to enhance analytical efficiency. One study utilizing the Aeonose™ device demonstrated a high sensitivity of 94.4% and a negative predictive value (NPV) of 85.7% for detecting non-small cell LC. Furthermore, the Aeonose™ exhibited the ability to distinguish between different LC subtypes, including adenocarcinoma and squamous cell carcinoma, with varying degrees of sensitivity and specificity [22].

## Working Principle of E-Nose

An E-nose aims to replicate the principles of smell, offering a different perspective on the volatile compounds compared to its biological counterpart [14]. VOCs are the key detectors/key factors in a patient, which are generated by various metabolic pathways. Due to exposure to high-risk carcinogens like tobacco smoking will produce VOC by 2 ways, the first way is these generated carcinogens synthesizes free radicals and reactive oxygen species (ROS), which contain an unpaired electron in their outer shell, can originate from both endogenous and exogenous sources, such as cigarette smoke [23]. These reactive molecules interact with proteins and fatty acids, leading to the formation of VOCs [24]. The second way by which VOC can be produced is by induction of cytochrome P450 enzymes (a group of oxidase enzymes). In a healthy individual, the cytochrome P450 enzyme is not induced so this enzyme clears the VOC in the body but in a LC patient clearance of VOC like alkane and aldehyde is altered.

The major VOCs associated with LC, as reported in the literature, include 2-butanone, which is frequently highlighted as a significant biomarker, along with 1-propanol, isoprene, ethylbenzene, and styrene, the latter two being increasingly recognized in recent studies. Additionally, compounds such as hexanal, acetone, carbon disulfide, dimethyl sulfide, and 2-pentanone have been identified in LC patients [25]. Furthermore, butadiene, orotic acid, tetrahydrobiopterin, and N-phenylacetylglutamine have been found to exhibit significant differences in concentration when comparing LC patients to healthy controls, suggesting their potential role in disease identification and biomarker development [26]. Along with VOC, non-volatile compounds like conjugated dienes and malondialdehyde are also generated.

So, this gives us a clear idea that VOCs are present only in LC patients as they undergo some metabolic pathways. To detect the VOC in earlier studies, show that using canine dog olfactory system, the difference between breath sample of lung and breast cancer patient to healthy patients was done [27].

**Fig. 2:**
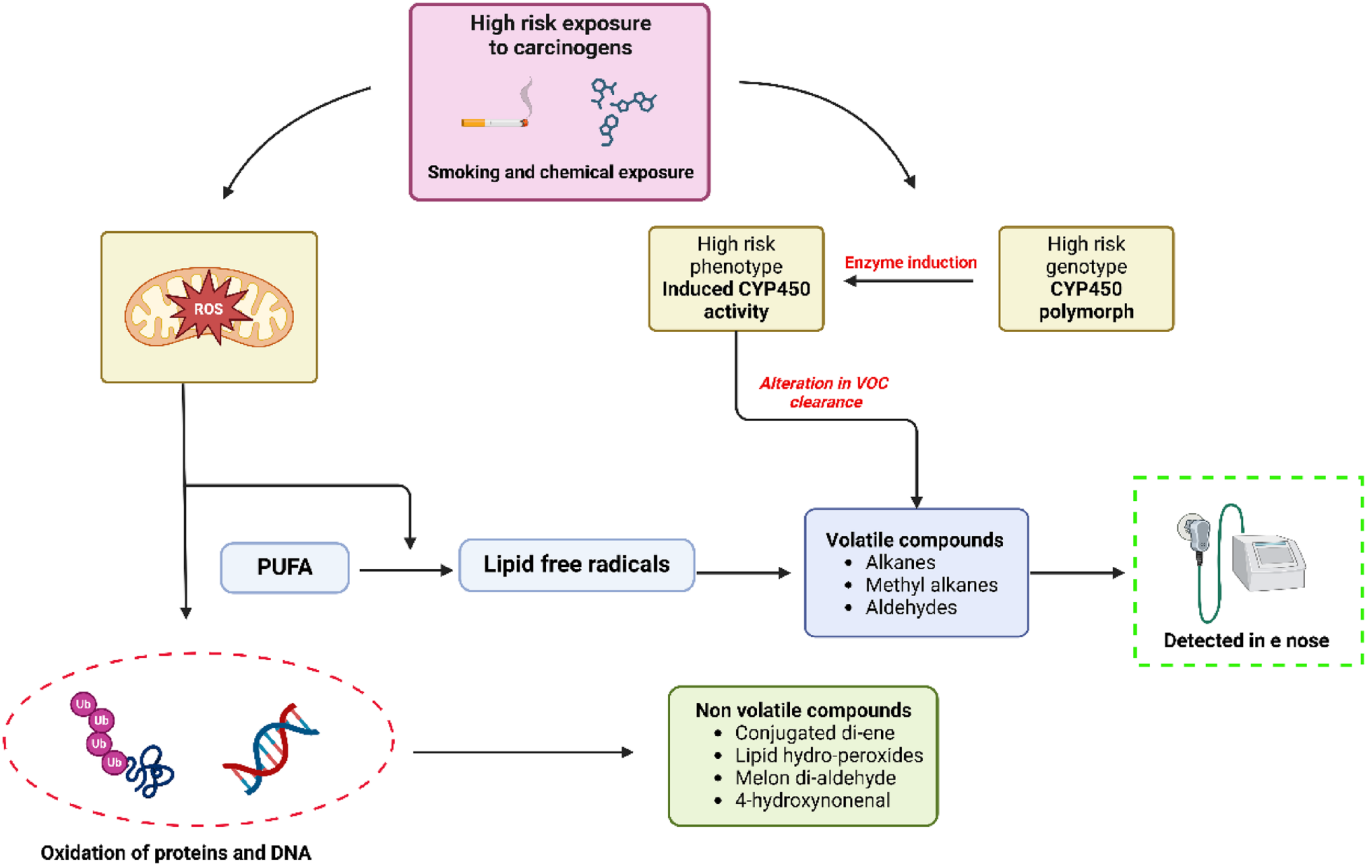
The metabolic pathway leading to generation of VOC; PUFA: Poly-unsaturated fatty acids; ROS: Reactive oxygen species; CYP450: Cytochrome P450

The exhaled VOC can be analyzed by either VOC identification or VOC patterning. VOC identification is the characteristic biomarker associated with the disease identified. The principle involved in this is gas chromatography coupled with mass spectroscopy (mass-to-charge ratio). Although quantitative analysis using GC–MS has not consistently enabled reliable early detection of lung cancer, it plays a crucial role in identifying potential breath biomarkers and constructing population-specific VOC libraries. These libraries are foundational for training E-nose systems in pattern recognition, enabling the device to distinguish between healthy and disease-related breath profiles [28, 29]. The metabolic pathway leading to the generation of VOC is summarized in Fig. 2. The sensors used are selective sensors, and VOC patterning is the exhaled VOC fingerprint that is observed. This aims at defining specific patterns of disease-related VOC. The sensor used here is cross-relative VOC.

There are different steps involved in the detection of LC by E-nose as discussed below:

### Sample Collection

Exhalate or breath sampling is a critical step in E-nose-based diagnostic workflows, significantly influencing the accuracy and reliability of sensor responses. Commonly used techniques include

1. **Direct Exhalation into Sensor Chamber**: In this approach, the individual breathes directly into a sensor-equipped chamber. The structure of the chamber plays a vital role in maintaining consistent airflow and enhancing the capture of exhaled compounds. Techniques such as Computational Fluid Dynamics (CFD) can aid in refining the chamber design to improve sensor performance [30, 31].
2. **Breath Sample Bags (e.g., Tedlar® Bags)**: Breath samples may be gathered using collection bags like Tedlar® and subsequently transferred to the sensor chamber for examination. This technique enables both storage and delayed analysis of the samples, making it advantageous for a range of diagnostic uses [32].
3. **Mask and Tube-Based Collection Systems**: An innovative method incorporates the E-nose sensor array directly into a face mask, enabling real-time breath analysis during wear. This design offers a practical solution for continuous, non-invasive monitoring, with potential applications in areas like respiratory health assessment and alcohol level detection. Alternatively, tube-assisted systems can channel exhaled breath from the individual to the sensor chamber. This approach ensures efficient sample delivery with minimal risk of loss or external contamination, thereby maintaining the integrity of the collected breath sample [32].
4. **Condensate or Filter-Based Collection**: This technique captures VOCs from exhaled breath by collecting condensate or employing specialized filters. The gathered material is subsequently examined using the E-nose system. This method is especially effective for isolating targeted biomarkers present in the breath [32, 33].

### Sensor Response and Signal Processing/Analyte Identification

The sensor array is the combination of several different gas sensors with a microfabrication process (MEMS) [34]. Each sensor in the array behaves like a receptor responding to different odors to varying degrees. When the sensor array is exposed to an odor, the sensors interact with the VOCs, causing changes in their physical properties, such as electrical resistance. These changes are converted into electrical signals that represent the odor’s unique “smell print” [35]. Identification and classification of an analyte mixture are accomplished through recognition of this unique aroma signature (electronic fingerprint) of collective sensor responses. A reference library of digital aroma patterns for known samples is built before analyzing unknowns. The artificial neural network (ANN) is trained using pattern recognition algorithms to distinguish between analyte types, continuing until the desired level of differentiation is reached. The results are validated and added to the library, which is then used to compare with unknown samples. Identification of unknowns relies on the shared aroma attributes between their patterns and those in the library [36].

### Pattern Recognition

**Fig. 3:**
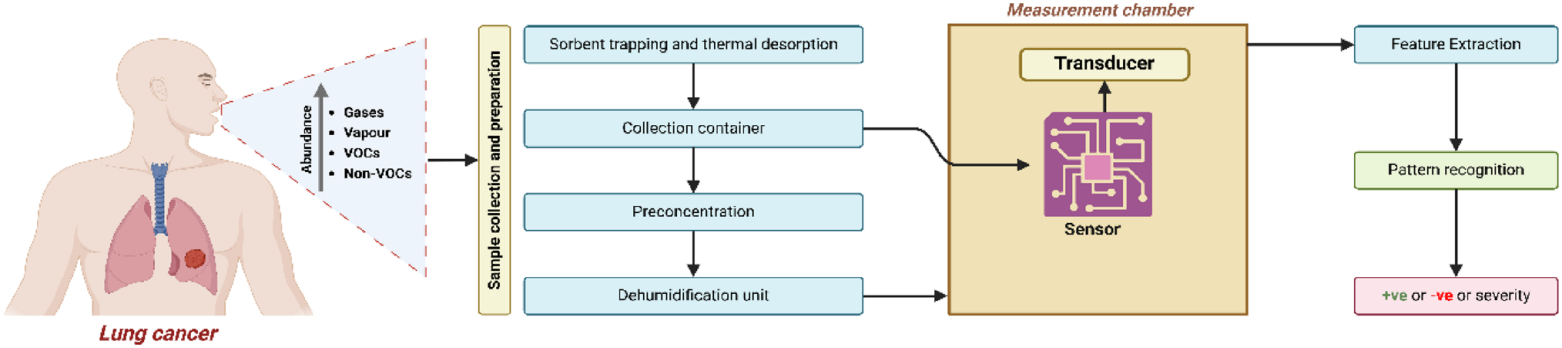
Stepwise process of breath analysis involving sample collection and preparation, dehumidification, and pattern recognition

Interface circuits process and convert sensor signals into electrical signals, finally the recognized results are obtained with various recognition algorithms [37] such as principal component analysis (PCA), cluster analysis, artificial neural networks (ANN), and support vector machines (SVM), are used to analyze the sensor data and identify odor patterns [38]. The stepwise process of breath analysis is shown in Fig. 3.

## Sensors

The first gas multisensory array was invented in 1982. The core of an E-nose is an array of chemical sensors, each with partial sensitivity to a range of gases rather than a specific one. These sensors can include metal oxide semiconductors, optical sensors, amperometric gas sensors, surface acoustic sensors, and piezoelectric gas sensors [39].

1. **Metal Oxide Semiconductor (MOS) Gas Sensor**

In 1968, Taguchi introduced the first commercially available MOS gas sensor [40], which features a ceramic cylinder (9.5 mm in length, 3 mm in diameter) with a heating coil and a metal oxide coating, typically stannic oxide (SnO_2_) doped with palladium or platinum, although other oxides like Zinc oxide (ZnO), tungsten trioxide (WO_3_), and titanium dioxide (TiO_2_) can also be used. The sensor's resistance changes when exposed to VOCs or gases such as nitric oxide (NO) due to redox reactions: during the oxidizing phase, oxygen adsorbs onto the surface, trapping electrons and increasing resistance, while in the reducing phase, oxygen reacts with VOCs, releasing the trapped electrons and lowering resistance. The extent of resistance change depends on the type of VOC and the grain size of the metal oxide. Selectivity can be enhanced by modifying the catalyst or adjusting the sensor's operating conditions. MOS sensors typically operate at temperatures between 300 and 500 °C, facilitating rapid and reversible reactions on the surface while preventing the formation of chemisorbed water, which could impede the reaction with VOCs [41].

2. **Optical sensors**

Optical sensors are highly versatile because they enable the simultaneous capture of both intensity and wavelength data, utilizing various techniques such as absorbance, reflectance, fluorescence, refractive index, and colorimetry [40]. Optical sensors play a significant role in E-nose technology by detecting VOCs through interactions with light [42]. These sensors offer high sensitivity, fast response times, and the ability to operate at room temperature, making them attractive for breath analysis in LC detection [35]. In optical sensors, there are two analyzed mediums: gas and liquid; sensing materials in gas are fluorescent dyes and metalloporphyrin, which detect VOC such as toluene, ethanol, methanol, and acetone [43], whereas sensing materials in liquid are fluorescent dyes and aptamers, which are useful in protein detection [44–46].

3. **Piezoelectric gas sensors**

Piezoelectricity is a phenomenon observed in certain anisotropic crystals, where the application of mechanical stress induces the formation of electric dipoles. In the context of gas sensing, piezoelectric sensors operate based on mass change detection [47]. When VOC molecules adsorb onto the sensor's surface, they alter its mass, leading to a shift in its resonant frequency [48]. This frequency shift serves as a measurable parameter to determine VOC concentration. Quartz and lithium niobate (LiNbO₃) are among the most widely used piezoelectric crystals for gas detection [49]. The two primary types of piezoelectric sensors employed in E-nose technology are bulk acoustic wave (BAW) and surface acoustic wave (SAW) sensors.

4. **Polycarbonate (PC) sensors**

Polycarbonate (PC) serves as an advanced smart sensor material for detecting VOCs through resistive band intensity ratios in the white light reflection spectrum. PCs, as periodic structures in one, two, or three dimensions, control electromagnetic wave propagation via photonic band gap formation, which arises from refractive index contrast between constituent materials. The incorporation of cavities in PC structures enables localized resonant modes for gas and liquid sensing applications. Additionally, PC-based colorimetric sensors overcome photobleaching issues, enhancing sensor longevity. PC sensors exploit uniform pore structures and large surface areas to achieve high sensitivity. The integration of inorganic and organic materials within PCs enhances photonic stop band shifts, improving sensor performance. Despite these advantages, PC sensors face challenges related to high fabrication costs, complex manufacturing processes, and structural fragility, necessitating further research to optimize optical stability, transmission quality, and commercial viability [50]. Comparative analysis of technology readiness and clinical applicability of e-nose systems for lung cancer detection is summarized in Table 1.

**Table 1:** Comparative analysis of technology readiness and clinical applicability of e-nose systems for lung cancer detection

S. No.	Aspect	Technology readiness	Clinical applicability	References
1	Early-Stage Detection	E-nose shows high accuracy and strong *F*1 scores (balance of sensitivity and precision) in detecting stage I lung cancer	Effective in distinguishing early-stage lung cancer from benign nodules	[51]
2	Differentiation of diseases	High precision in differentiating lung cancer from COPD using machine learning	Shows potential as a prognostic and diagnostic tool for lung cancer in patients with COPD	[52]
3	Sensor technology	Utilizes metal oxide and surface acoustic wave sensors known for high sensitivity to VOCs	Effective in detecting VOCs specific to lung cancer	[53]
4	Machine learning integration	Advanced algorithms enhance sensitivity and specificity	Machine learning enhances diagnostic accuracy, reducing false positives, and improving clinical decision-making	[54]
5	Non-invasive testing	E-nose provides a non-invasive method for lung cancer detection	Promising for non-invasive, point-of-care screening, and early lung cancer diagnosis, especially in high-risk populations	[55]
6	Clinical trials and studies	Multiple studies and trials demonstrate high diagnostic performance	Multiple trials have demonstrated high sensitivity and specificity in real-world clinical settings	[51, 52]

## Selectivity and Sensitivity of E-Nose Sensors

The performance of an E-nose largely depends on two critical parameters: selectivity and sensitivity. Selectivity refers to the sensor’s ability to distinguish between different gases or VOCs, while sensitivity represents its capacity to detect even minute concentrations of target analytes. Both factors play a crucial role in determining the effectiveness of E-nose systems.

## Sensitivity of E-Nose Sensors

Sensitivity defines the lower detection limit (LOD) of a sensor, which is the minimum concentration of an analyte that can be reliably detected. Various factors influence sensitivity, including the sensor material, operating temperature, and environmental conditions such as humidity. Different sensor technologies offer varying levels of sensitivity:

***Surface Acoustic Wave (SAW) Sensors***: SAW sensors, particularly uncoated ones, exhibit high sensitivity, stability, low noise, and long lifetimes, making them ideal for applications like breath diagnosis. Their sensitivity can be further enhanced by polymer coatings such as OV-101, PEG-1540, and OV-17, which improve detection of specific VOCs [56].

***Quartz Crystal Microbalance (QCM) Sensors***: The sensitivity of QCM sensors is proportional to the square of their resonant frequency, meaning higher frequencies (e.g., 20 MHz) result in greater sensitivity compared to lower frequencies (e.g., 12 MHz). Among various sensing films tested, ethyl cellulose was found to be the most sensitive [57].

***Metal Oxide Semiconductor (MOS) Sensors***: MOS sensors, such as those based on TiO₂, exhibit high sensitivity even at room temperature, particularly for gases like hydrogen. However, their performance can be influenced by humidity [58].

***Surface Plasmon Resonance (SPR) Sensors***: SPR sensors, especially those utilizing multilayer black phosphorus (BP), offer enhanced sensitivity and high detection accuracy for various toxic and flammable gases [59].

## Selectivity of E-Nose Sensors

While high sensitivity is desirable, selectivity ensures that a sensor responds primarily to the target gas without interference from other compounds. Achieving good selectivity is challenging because most gases have overlapping chemical properties. Various strategies have been explored to enhance selectivity, including temperature modulation, sensor array optimization, pressure modulation, and advanced computational techniques such as machine learning (ML) [60]. A recent study demonstrated a data-driven approach to improving selectivity in an AZO-based E-nose by integrating ML and principal component analysis (PCA). The sensor array was exposed to a series of structurally similar alcohols, including methanol, ethanol, 1-propanol, 2-propanol, 1-butanol, and isobutanol, generating distinct resistance-based “fingerprints” for each analyte. PCA was employed to reduce the dimensionality of the sensor data, mapping analyte responses into a feature space where they formed well-separated clusters. This transformation allowed for enhanced discrimination between analytes with minor structural differences.

To further refine selectivity, the sensor response patterns were cross-referenced with molecular fingerprints obtained from the PubChem database. A Decision Tree regression model was then used to establish correlations between sensor responses and molecular structures, enabling the system to differentiate between close homologs and isomers without requiring prior training data. This AI-driven approach demonstrated that by leveraging chemical space mapping, an E-nose system could achieve high selectivity without the need for predefined reference datasets.

These findings highlight the potential of self-learning gas sensing methodologies in overcoming conventional selectivity limitations. The integration of sensor response modeling with molecular databases and ML algorithms offers a promising path toward intelligent E-nose systems capable of precise analyte differentiation, even in complex chemical environments. Such advancements pave the way for next-generation E-nose technologies with improved adaptability and specificity, making them invaluable for applications in medical diagnostics, environmental monitoring, and industrial quality control [61].

Accuracy, sensitivity, and specificity are key metrics used to evaluate the performance of a classification system. Accuracy is calculated as [(number of true positive + number of true negative)*100/number of total populations], representing the overall correctness of the model. Sensitivity, given by [number of true positives*100/ (number of true positives + number of false negatives)], measures the ability to correctly identify positive cases for instance, the proportion of individuals with a disease who are accurately diagnosed. Specificity, calculated as [number of true negatives*100/ (number of true negatives + number of false positives)], determines how well the model identifies negative cases, such as the percentage of healthy individuals correctly classified as disease-free [62].

## Instrumentation and System Design

Gardner and Bartlet [36] outlined the fundamental components required for an E-nose device, which include

- A system for aroma delivery that transports volatile aromatic compounds from the sample source to the sensor array.
- A sensor chamber maintained at a stable temperature and humidity to prevent variations that could impact the adsorption of aroma molecules.
- An electronic transducer responsible for converting chemical signals into electrical signals, followed by amplification and conditioning.
- A digital converter that transforms the electrical (analog) signal into a digital format.
- A microprocessor that processes the digital signal, displays the results, and conducts statistical analysis for sample classification or identification.

## Components of E-Nose system

The E-nose majorly consists of sample inlet (Aroma Delivery System), sensor array chamber, gas sensors array, processing circuit, control circuit, and the PC for data analysis [63].

1. **Aroma delivery system**

The aroma delivery system is a crucial component of an E-nose, responsible for transferring volatile compounds from the sample source to the sensor array. The sample, consisting of gaseous or VOCs, is introduced into the system using a pump, fan, or diffusion-based mechanism, ensuring a steady and directed airflow toward the sensor chamber. This controlled airflow is essential to maintain uniform exposure of the sensors to the analytes, leading to consistent and reliable detection [64].

2. **Sensor array chamber**

The sensor chamber houses the sensor array and plays an important role in ensuring optimal sensor performance. Its design and structure directly influence key factors such as response time, gas flow distribution, and the contact area between gas molecules and sensors [65]. A well-designed chamber enhances detection accuracy, repeatability, and stability, making it a crucial aspect of E-nose functionality.

3. **Sensor array**

The sensor array is the central component of an E-nose, consisting of multiple gas sensors, such as metal oxide semiconductors, optical sensors, piezoelectric sensors, and chemiresistive sensors, designed to detect a wide range of VOCs [39]. Each sensor responds uniquely to different gases, producing a distinct signal pattern or “fingerprint” for each odor [66, 67].

4. **Processing unit**

By utilizing advanced computational techniques, the processing unit ensures accurate and efficient analysis, enabling real-time decision-making in various applications [68]. The performance of an E-nose system largely depends on the efficiency of its processing unit, which integrates machine learning algorithms, pattern recognition methods, and real-time computing capabilities [69]. With advancements in artificial intelligence, embedded computing, and edge processing, modern E-nose systems are becoming more sophisticated, offering improved accuracy, adaptability, and portability [70].

The following sections explore the key functions, importance, and technological advancements of processing units in E-nose systems.

- **Signal Processing**

The raw data collected from the sensor array undergo several signal processing steps to ensure accuracy and reliability. These steps include noise reduction, baseline correction, drift compensation, and normalization to standardize the data for further analysis. Advanced filtering techniques, such as wavelet transforms and Kalman filtering, can be employed to improve signal quality and mitigate environmental interference [71].

(b) **Pattern Recognition**

To extract meaningful information from sensor responses, various pattern recognition algorithms are implemented. Traditional methods like Principal Component Analysis (PCA) and Linear Discriminant Analysis (LDA) help in dimensionality reduction and feature extraction [72], whereas more complex techniques such as Decision Trees, k-Nearest Neighbors (KNN), and Artificial Neural Networks (ANN) enhance classification accuracy [68, 72]. Recent advancements also include ensemble learning approaches, such as random forests and boosting algorithms, to improve robustness in odor classification.

(c) **Data Analysis**

Machine learning and deep learning techniques have significantly enhanced the performance of E-nose systems. Convolutional Neural Networks (CNN) are widely used for feature extraction and classification, particularly in real-time applications [73]. Additionally, recurrent neural networks (RNNs) and long short-term memory (LSTM) networks are being explored for analyzing time-series sensor data, enabling improved pattern recognition in dynamic environments. The integration of online learning algorithms allows continuous system updates, enhancing adaptability in changing conditions [68]. Furthermore, advanced systems incorporate memory management strategies to prevent catastrophic interference, allowing the system to learn and adapt over time [74].

5. **Control unit**

The controller unit in an E-nose plays a crucial role in managing the overall functionality of the device. It handles tasks such as sensor data acquisition, signal processing, and communication with external devices [39]. It typically consists of a microcontroller or a digital signal processor (DSP) that performs multiple tasks, including sensor management, signal conditioning, control functions, and user interaction. By interfacing with gas sensors, the controller collects data and processes raw signals to enhance their suitability for further analysis. Additionally, it regulates the timing and sequence of operations, ensuring efficient data acquisition, storage, and transmission [75]. Moreover, the controller may also facilitate user interaction by displaying gas concentration values and triggering alarms when necessary [76].

**Fig. 4:**
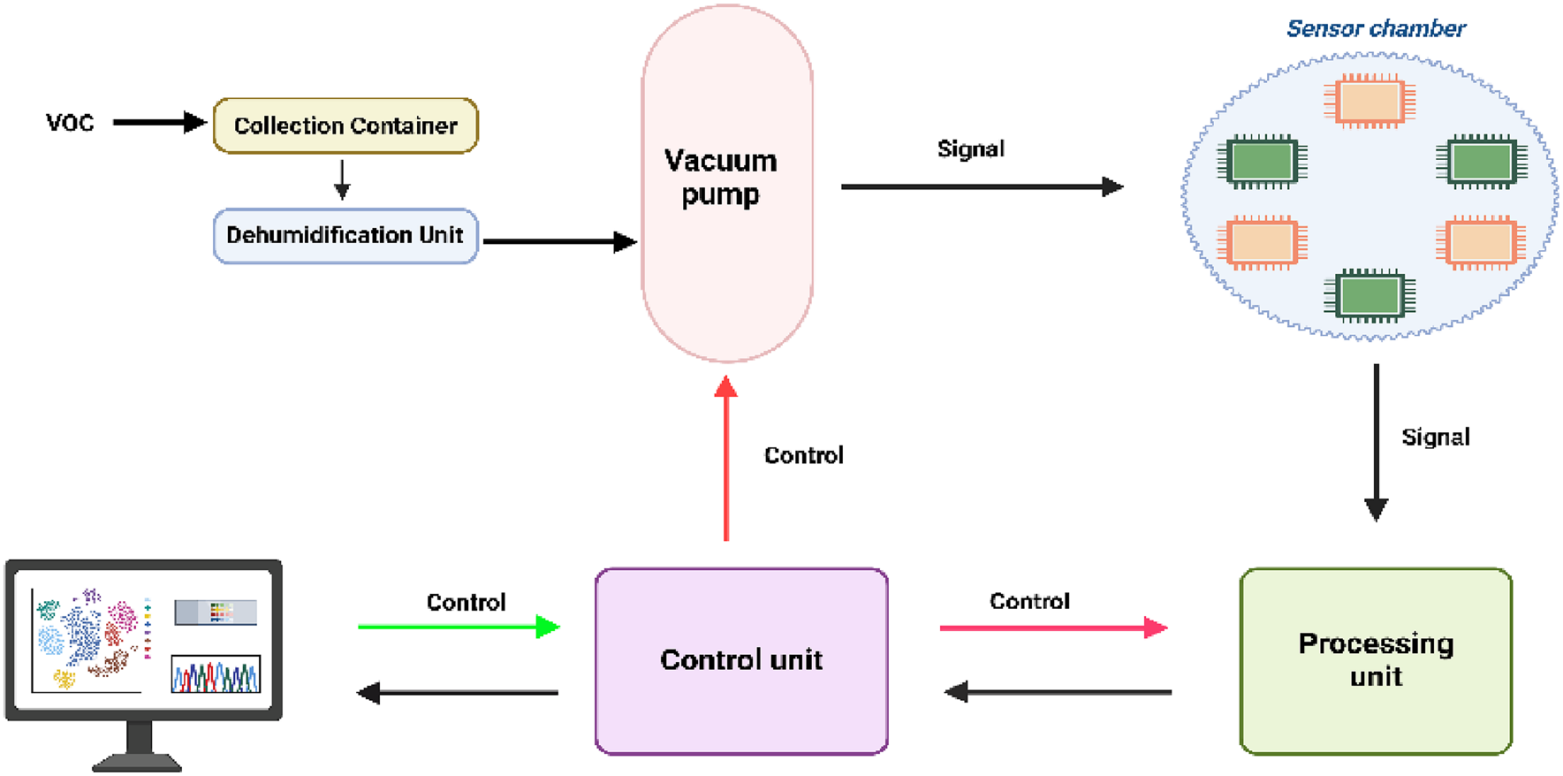
Schematic Representation of an Electronic-Nose System

The processing unit can either be embedded within the microcontroller or function as a separate module dedicated to handling complex computations. In the E-nose system, the Arduino Uno, based on the ATmega328P microcontroller, is commonly used for control functions due to its efficiency and reliability. It features a resettable polyfuse for USB port protection and operates at 5 V, with a recommended input of 7 V to 12 V. Unlike earlier models, it utilizes a programmed ATmega16U2 for USB-to-serial conversion. With 14 digital Input/output pins, a 16 MHz clock speed, and 32 KB flash memory, the Arduino Uno effectively handles data acquisition and system control [63]. Other examples of microcontrollers utilized in E-noses include mbed microcontrollers based on ARM architecture and C8051F020 System-on-Chip (SoC) devices [77]. The schematic representation of an E-nose System is depicted in Fig. 4.

## Applications of E-noses in LC

The LC detection through E-nose is by non-invasive technique, it not only helps in early detection of LC but also helps in differentiation of LC with other chronic pulmonary diseases.

1. **Breath Analysis: A Non-Invasive Approach for Early detection of LC**
2. Diagnosis of LC using E-nose (discriminating LC patients from healthy controls)

Recent advancements in E-nose technology have significantly improved LC detection and monitoring. Mainardi et al. developed an E-nose with a metal oxide sensor array and an artificial neural network (ANN), validated through leave-one-out cross-validation, which demonstrated 85.7% sensitivity, 100% specificity, and 93.8% accuracy in distinguishing LC patients from healthy individuals [78].

Further research involving 114 LC patients and 147 healthy controls utilized an E-nose with metal oxide semiconductor gas sensors and machine learning algorithms, where the XGBoost classifier achieved 91.67% accuracy in training and 83.33% sensitivity with 86.27% specificity in validation, reinforcing its potential as a non-invasive diagnostic tool [79]. Traditional E-nose models often struggle with accuracy, high false-negative rates, and limited generalizability. Addressing these issues, the PCA-SVE framework combines Principal Component Analysis for feature extraction with an ensemble learning approach that integrates SVM, decision trees, random forests, logistic regression, and K-nearest neighbor regression, enhancing detection performance [80].

Moreover, an optimized AdaBoost classifier incorporating genetic algorithms (GA) and heterogeneous sub-classifiers further improved accuracy (98.47%), sensitivity (98.33%), and specificity (97%), surpassing support vector machines, logistic regression, and random forests in stability and generalizability. However, challenges remain in distinguishing LC subtypes, necessitating larger and more diverse datasets for enhanced clinical applicability [81]. Beyond detection, E-nose technology has also shown promise in monitoring immunotherapy responses in NSCLC. Breath analysis using SpiroNose effectively differentiated responders from non-responders to anti-PD-1 therapy, achieving 100% sensitivity and 76% specificity in validation. The predictive power of the on-treatment model (six weeks post-therapy) was superior to baseline measurements, with an AUC of 0.97, highlighting its potential for guiding personalized treatment strategies [82].

The standardized retention time for each alkane sample was converted into Kovats Indices (KI), a widely recognized chromatographic parameter that ensures the reproducibility and comparability of VOC identification across different analytical systems, enabling precise differentiation of VOCs between LC patients and healthy individuals. This method minimizes retention time variability due to instrumental and environmental factors, enhancing the accuracy of compound identification. By utilizing KI, the study effectively standardized the detection of key biomarkers such as hexane and 2,2,4,6,6-pentamethylheptane, which exhibited elevated concentrations in LC patients, while 1,2,6-trimethylnaphthalene displayed reduced abundance, suggesting a protective role. The integration of KI-based profiling with stepwise regression analysis enabled the identification of statistically significant parameters influencing LC detection, culminating in the development of a logistic regression formula, *P* = 1/[1 + *e*^(−9.005+102*X1+0.011*X2+0.022*X3–0.517*X4)^], which incorporates age, hexane, 2,2,4,6,6-pentamethylheptane, and 1,2,6-trimethylnaphthalene. This formula provides a quantitative framework for assessing LC probability based on breath analysis, demonstrating the potential of chromatographic standardization combined with advanced statistical modeling in non-invasive cancer diagnostics [83]. Table 2 summarizes studies conducted on the use of E-nose technology for diagnosing LC, specifically in distinguishing LC patients from healthy controls. These studies highlight the effectiveness of E-nose sensors in detecting VOCs associated with LC.

**Table 2:** Summary of studies conducted to differentiate LC patients and healthy individuals using different E-nose technology

Study No.	Total participants	controls	LC patients	Sensor type	Specificity (%)	Sensitivity (%)	Accuracy	References
1	199	120	79	aeoNose + multivariable logistic regression analysis	49.2	94.7	AUC-0.86	[84]
2	16	10	6	metal oxide sensors array	100	85.7	93.8%	[78]
3	44	12	32	metal-doped WO3 nanowires	–	–	98.6%	[85]
4	261	147	114	Metal oxide semiconductor gas sensor + machine learning algorithm	86.27	83.33	91.67%	[79]
5	124	72	52	Z-nose4200 equipment	94.0	76.00	82.8%	[83]
6	130	90	40	MOS sensor	–	–	75.0% (k-nearest neighbors) 92.3% (cross-validation)	[86]
7	150	99	51	TGS gas sensor along with XG Boost method	–	–	LC-79.31% COPD-76.67%	[87]
8	62	40	22	TGS sensors + linear discriminant analysis, K-nearest neighbors, logistic regression	95.62	88.63	93.14% AUC-0.98	[88]
9	–	–	214	Gas sensors	96.96	94.78	95.75%	[80]
10	142	51	91	electrochemical sensors, metal oxide semiconductor, and controllable temperature sensor	97	98.33	98.47%	[81]
11	124	40	84	weighted discriminative extreme learning machine (WDELM)	81.5	87.26	81.5%	[2]
12	80	40	40	Quartz microbalance sensor + Gas chromatography-mass spectrometry (GC–MS)	63.6–75.0	50.0–69.2	–	[89]
13	–	40	24	AI based Metal oxide semiconductor + Random Forest, logistic regression (fivefold cross-validation)	–	–	Random forest-85.38% Logistic regression– 83.59%	[90]

(b) Differentiating LC from other chronic respiratory diseases using E-nose

E-nose technology, combined with advanced machine learning algorithms, has shown significant promise in the non-invasive detection and differentiation of respiratory diseases such as LC and COPD. Recent studies have demonstrated the efficacy of E-nose devices in analyzing exhaled breath to identify disease-specific VOCs.

For instance, a study involving 199 participants comprising 51 LC patients, 55 COPD patients, and 93 healthy controls utilized an E-nose system to analyze breath samples. Among the machine learning models applied, XGBoost outperformed others, achieving accuracies of 93.04% for LC detection and 90.68% for COPD detection. However, validation results indicated reduced accuracies (79.31% and 76.67%, respectively), likely due to sample size limitations. Receiver Operating Characteristic (ROC) analysis confirmed strong discriminative abilities, with AUC values of 0.94 for LC and 0.97 for COPD [87]. Another study assessed the clinical feasibility of an E-nose system with 32 LC patients, 38 COPD patients, and 72 healthy controls. The device's portability and robust design enhanced usability. For LC detection, the k-nearest neighbors algorithm achieved 91.3% accuracy, 84.4% sensitivity, and 94.4% specificity. In contrast, the support vector machine algorithm performed better for COPD detection, with 90.9% accuracy, 81.6% sensitivity, and 95.8% specificity [91]. A cross-sectional study analyzed exhaled breath from 30 LC patients, 50 breast cancer patients, 50 COPD patients, and 50 control subjects using an E-nose. Multivariate analyses, including PCA and canonical analysis of principal (CAP) coordinates, were conducted. PCA explained over 90% of the data and achieved 100% correct classification between patients and controls. CAP distinguished among the four groups with two canonical axes, achieving a 91.35% overall correct classification. The model accurately predicted 20 single-blind samples with 100% correctness [92].

In a study by Poli et al., GC–MS was utilized to analyze the chemical fingerprints of patients. The study achieved a discrimination accuracy of 72.2% for LC patients (*n* = 36) and 82.7% for COPD patients (*n* = 25). The analysis demonstrated a sensitivity of 72% and a specificity of 93.6% [24].

Furthermore, a study involving 84 COPD patients and 53 LC patients utilized E-nose technology combined with machine learning to analyze exhaled breath. The classification model achieved 94.16% accuracy, with sensitivity and specificity rates of 96.34% and 90.91%, respectively, and an AUC value of 0.91 [52]. These results highlight E-nose technology's potential in non-invasive respiratory disease diagnosis. Collectively, these studies underscore the potential of E-nose technology, particularly when integrated with sophisticated machine learning techniques, in accurately diagnosing and differentiating respiratory diseases such as LC and COPD. The high sensitivity, specificity, and overall accuracy reported in these studies suggest that E-nose devices could serve as valuable non-invasive diagnostic tools in clinical settings.

(c) Differentiation of various stages and types of LC using E-nose

The application of E-nose technology in LC diagnosis extends beyond detection to staging and differentiation of cancer types. This study explores its effectiveness in distinguishing early LC stages (I & II), though a key limitation is the scarcity of early-stage samples due to subtle symptoms, which impacts diagnostic accuracy. Additionally, the limited diversity of collected data sourced from only two hospitals may restrict the system’s generalizability [93]. Supporting the potential of E-nose as a non-invasive diagnostic tool for differentiating type of LC, a study by Tarik Saidi and colleagues identified four novel LC biomarkers and demonstrated high diagnostic accuracy. Their findings revealed an overall accuracy of 98.6% in LC detection, 84.5% accuracy in distinguishing small cell LC (SCLC) from non-small cell LC (NSCLC), and 77.5% accuracy in differentiating NSCLC subtypes, such as squamous cell carcinoma (SCC) and adenocarcinoma (ADC) [85]. These results highlight the promising role of E-nose technology in both LC staging and subtype differentiation, reinforcing its potential as a complementary diagnostic approach.

In all the aforementioned studies and research, LC detection using E-nose relies solely on analyzing the individual's exhaled breath, making it a completely non-invasive diagnostic method. Breath collection is effortless, painless, and does not require any surgical or intrusive procedures, ensuring patient comfort and ease of sampling. By detecting specific VOCs in breath, E-nose technology provides a promising, rapid, and patient-friendly approach for early LC detection. Furthermore, no adverse effects were observed in any subject as a result of providing a breath sample, confirming that this method is not only non-invasive but also safe and well-tolerated for repeated use in clinical and screening settings [81, 83]. It may be particularly useful in high-risk populations and in patients where biopsy is risky or challenging [51].

2. **Clinical Trials and Approved E-nose Devices for LC Diagnosis**

In a Phase IIc, clinical trial (NCT04734145) on 100 patients diagnosed with a ≥ 50% solid stage I lung nodule evaluated the performance of E-nose technology in LC detection. The E-nose correctly classified 86% of cases, achieving an F1 score of 92.5%, which demonstrated strong agreement with histopathologic results [51]. In other studies, 893 patients, including 682 patients with COPD and 211 patients with LC, were analyzed in the study. Principal Component 3 with a p value of less than 0.001 showed a significant difference between early-stage (I and II) and advanced-stage (III and IV) LC. The E-nose technology successfully distinguished LC stages with a cross-validated accuracy of 88% and AUC of 0.93, with a confidence interval ranging from 0.87 to 0.98, highlighting its potential for staging LC non-invasively [94]. A clinical study on Aeonose® (NCT02951416) demonstrated 84% sensitivity, 97% specificity, and an AUC of 0.92 in detecting lung cancer via exhaled VOCs. However, in blinded validation, it misclassified 65.5% of remission patients and 11 of 24 COPD patients as positive, despite few later developing the disease [95].

The Lung Cancer Indicator Detection (LuCID) study is a multi-center trial analyzing breath samples from up to 2,500 patients to detect lung cancer via VOC profiling using GC–MS and GC-FAIMS. Findings will assess test accuracy, potentially enabling early detection and large-scale screening to reduce lung cancer morbidity and mortality [96]. The study, registered in The Netherlands Trial Register (No.: NL7025), comprised 376 participants in the training set and 199 in the validation set. A multivariable analysis based solely on clinical factors, including sex, age, and smoking history (pack-years), yielded an AUC-ROC of 0.67 for the training set and 0.75 for the validation set. Subgroup analysis indicated that the E-nose system maintained consistent performance across various LC stages, histological types, age groups, and sexes. Sensitivity and negative predictive value (NPV) were notably high in both early-stage (94 and 97%) and advanced-stage LC (84% and 90%). Furthermore, a comparison with the Mayo Clinic nodule calculator, which incorporates clinical and imaging parameters, showed that the breath-based model demonstrated higher sensitivity (94.6% vs. 83.8%) but lower specificity (22.2% vs. 61.1%). Despite this limitation, the breath analysis system exhibited strong potential as a non-invasive screening tool, particularly for ruling out LC in suspected cases [84]. Currently, there is no specific mention of an officially approved E-nose device for LC detection. However, several studies and developments indicate significant progress and potential in this area as it is mentioned above.

## Challenges, Solutions, and Future Directions

E-noses analyze VOCs in breath for non-invasive, rapid, and cost-effective LC detection. However, their clinical implementation faces several challenges related to accuracy, sensor stability, and environmental factors. Addressing these limitations requires advancements in sensor technology, machine learning, and standardized protocols. The following sections discuss the key limitations, potential solutions, and future advancements in E-nose technology for LC detection.

### Pre-Sampling Conditions and Their Impact on Test Accuracy

Patients were instructed to refrain from smoking, brushing their teeth, and consuming food or drinks for at least eight hours before sample collection [51]. Additionally, they were advised to avoid strong-smelling foods such as leeks, onions, pickled cabbage, garlic, and cabbage one day before the test [83]. Failure to adhere to these guidelines, whether accidentally or otherwise, could lead to inaccurate results. Moreover, maintaining such strict conditions solely for detection purposes can be challenging.

### Standardization, Biological Variability, and Regulatory Challenges in Breath Analysis

There is considerable variability in methodologies across breath analysis studies, including differences in patient selection, breath collection protocols, and data handling, which undermines reproducibility and reliability [97]. Diverse sensors and analytical techniques further contribute to inconsistent sensitivity and specificity [98]. Additionally, VOC profiles can vary significantly due to individual differences in diet, comorbidities, and environmental exposures, introducing biological noise that complicates biomarker discovery and model generalizability. Variations in respiratory patterns, expiratory/inspiratory flow rates, and upper-airway resistance can also significantly influence VOC concentrations, further complicating the interpretation of results [99]. Comorbid conditions such as COPD, asthma, and other metabolic or respiratory diseases may produce overlapping VOC signatures, making it challenging to isolate lung cancer-specific markers [91]. For instance, conditions like COPD or cardiovascular diseases can mask lung cancer symptoms, extending the diagnostic interval by 31–74 days [100]. Currently, no universally accepted standard exists for breath collection, storage, and analysis, hindering inter-study comparability. Regulatory approvals such as FDA clearance and CE marking demand robust evidence from large-scale, multicentre validation trials that are largely lacking. Most e-nose studies for lung cancer detection remain small feasibility trials with high risks of bias, insufficient for regulatory submission [54, 97].

To overcome these challenges, developing universal standards and quality control protocols through collaboration among researchers, industry, and regulators is critical. Early engagement with regulatory agencies can facilitate efficient trial design. Future efforts should prioritize large, well-designed clinical trials with diverse cohorts, rigorous validation, and AI-driven analysis to improve diagnostic accuracy and reproducibility. These steps are essential to achieve regulatory approval and enable clinical deployment of breath diagnostics as reliable, non-invasive tools.

### Sensor Drift and Environmental Influences

Drift refers to the slow degradation of sensor materials over time due to factors like humidity, temperature, pressure, and aging. This gradual decline affects the sensor’s long-term stability, leading to variations in readings even when exposed to the same VOCs [101, 102]. To mitigate this, maintaining consistent humidity, temperature, and pressure within the sensor chamber is essential. Additionally, future advancements could involve integrating multiple sensors with varying drift characteristics and utilizing modern sensor materials, such as self-regenerating metal oxides, to enhance performance. Disturbance refers to the interference caused by non-target VOCs, which can significantly affect sensor measurements. The techniques, like baseline correction and disturbance recognition, help mitigate these effects by removing unwanted compounds [101].

### Contamination Challenges in Breath Collection

Exhalate collection is prone to contamination from environmental factors, background VOCs, and improper handling, all of which compromise E-nose accuracy. Interference and cross-sensitivity remain major challenges, especially in complex breath matrices [103]. Solutions include interference suppression techniques (e.g., PCA), controlled sampling conditions, advanced adaptive sensors, and standardized handling protocols [104].

### Integration and Portability Challenges

One of the main obstacles to clinical adoption of E-nose technology is miniaturizing and integrating its components into a compact, portable, and user-friendly design. Real-time breath analysis in point-of-care or home settings demands minimal infrastructure, but most current systems are bulky and lab-dependent. Miniaturization introduces challenges such as maintaining sensitivity and selectivity, ensuring stable signal processing, and enabling wireless data transmission, all crucial for making E-nose devices suitable for practical, real-world healthcare use [91].

Recent developments in nanomaterials, micro-electro-mechanical systems (MEMS), and system-on-chip (SoC) architectures are enabling more compact and power-efficient E-nose designs [105]. Future research should focus on optimizing sensor fabrication for small-scale formats, improving battery efficiency, and validating miniaturized E-nose systems in large clinical trials to ensure their reliability and scalability for widespread use.

### Multiomics Integration and Economic Considerations

The integration of E-nose technology into a multiomics diagnostic framework presents several key challenges and opportunities. Firstly, E-noses are often considered standalone tools. However, to improve diagnostic accuracy, they should be combined with other technologies like genomics, proteomics, metabolomics, breathomics, and imaging [106]. This combined approach offers a more complete picture of lung cancer and can help in early detection and better treatment planning. The solution is to build diagnostic models that include E-nose data along with other biological inputs and validate them using clinical studies.

Cost is also a major concern. While E-nose devices are affordable individually, integrating them with AI and multiomics platforms can become expensive. This includes costs for sensors, software, data storage, and maintenance [107]. To solve this, future research must include cost-effectiveness and cost–benefit studies. These studies should compare the total costs of E-nose-based systems with traditional methods and highlight potential long-term savings through early diagnosis and reduced need for invasive tests.

### Variability in Diagnostic Accuracy and Detection Thresholds

The accuracy of E-noses in detecting LC varies significantly, with sensitivity ranging from 48.3% to 95.8% and specificity from 10.0% to 100.0%, leading to potential false positives and negatives that complicate clinical decision-making [97]. Additionally, E-noses often require biomarker concentrations higher than those naturally present in breath samples to reliably differentiate between healthy and cancerous conditions [108]. This challenge can be addressed by developing standardized protocols for sample collection, preprocessing, and calibration. Future advancements include integrating E-nose data with other diagnostic tools or utilizing ultra-sensitive nanomaterial-based sensors, such as graphene and metal–organic frameworks, to enhance detection accuracy. A major limitation of current studies is their focus on advanced-stage cancer and small sample sizes, resulting in sample heterogeneity and potential confounding factors [109]. To address this, future research should prioritize early-stage cancer detection and include larger patient cohorts to accurately identify distinct breath prints associated with various cancer subtypes.

### Limitations of Single-Model Machine Learning Approaches

Relying on a single machine learning method can result in low detection accuracy and high false-negative rates. This challenge can be mitigated by implementing ensemble learning frameworks that integrate multiple high-performing models, such as decision trees, random forests, and support vector machines, to enhance overall accuracy, sensitivity, and specificity [110].

## Conclusion

E-noses have emerged as a promising, non-invasive tool for LC detection by analyzing VOCs in exhaled breath. Their functionality relies on VOC identification and pattern recognition, utilizing various sensor technologies such as metal oxide semiconductors, optical sensors, piezoelectric sensors, and polymer-based sensors. Machine learning algorithms, including support vector machines, artificial neural networks, and principal component analysis, enhance the system’s ability to differentiate between LC and other pulmonary diseases. The instrumentation and system design of E-noses consist of key components such as an aroma delivery system, sensor array chamber, processing unit, and control unit. The integration of microcontrollers, signal processing circuits, and advanced computing enables real-time analysis and improved detection accuracy. However, challenges remain, including sensor drift, environmental interference, and the need for stringent pre-sampling conditions. To overcome these issues, advancements in self-regenerating sensor materials, multi-sensor fusion, and improved calibration techniques are being developed.

In applications, E-noses have shown potential not only in detecting LC but also in differentiating it from other chronic respiratory diseases like COPD. Additionally, clinical trials have demonstrated their ability to classify LC subtypes and monitor treatment responses. Despite these advancements, most studies focus on advanced-stage cancer, necessitating further research on early detection and larger patient cohorts. Future developments in miniaturized, portable, and wireless E-noses, combined with hybrid diagnostic approaches and AI-driven analytics, will enhance their clinical reliability. Specific next steps should include the development of portable, low-cost E-nose devices suitable for use in primary care and resource-limited settings, along with the development of standardized protocols for breath sampling and analysis to ensure consistency and reliability. Integration of E-nose data with patient clinical information and electronic health records can enhance diagnostic accuracy and support personalized healthcare. Prospective, multicentre validation studies involving diverse populations are essential to establish generalizability and clinical utility. Moreover, expanding research beyond lung cancer to other diseases with VOC biomarkers will broaden the clinical applications of E-nose technology. Finally, combining E-nose outputs with imaging techniques or molecular biomarkers could improve diagnostic precision and enable a comprehensive assessment of lung cancer. With continued improvements, E-noses could revolutionize LC screening, offering a cost-effective, accessible, and accurate diagnostic solution for widespread medical use.

## Data Availability

No datasets were generated or analyzed during the current study.

## Abbreviations

LC: Lung Cancer
VOC: Volatile Organic Compounds
E-nose: Electronic Nose
AI: Artificial Intelligence
CT: Computer-based Tomography
GC-MS: Gas Chromatography-Mass Spectrometry
NPV: Negative Predictive Value
ROS: Reactive Oxygen Species
CYP450: Cytochrome P450
PUFA: Poly-unsaturated Fatty Acids
MEMS: Micro-Electro-Mechanical Systems
ANN: Artificial Neural Network
PCA: Principal Component Analysis
SVM: Support Vector Machine
MOS: Metal Oxide Semiconductor
SnO₂: Stannic Oxide
ZnO: Zinc Oxide
WO₃: Tungsten Trioxide
TiO₂: Titanium Dioxide
SPR: Surface Plasmon Resonance
BAW: Bulk Acoustic Wave
SAW: Surface Acoustic Wave
PC: Polycarbonate
MOF: Metal-Organic Framework
QCM: Quartz Crystal Microbalance
ML: Machine Learning
AUC: Area Under Curve
DSP: Digital Signal Processor
CNN: Convolutional Neural Network
RNN: Recurrent Neural Network
LSTM: Long Short-Term Memory
SoC: System-on-Chip
NSCLC: Non-Small Cell Lung Cancer
SCLC: Small Cell Lung Cancer
SCC: Squamous Cell Carcinoma
ADC: Adenocarcinoma
COPD: Chronic Obstructive Pulmonary Disease
GC-FAIMS: Gas Chromatography—Field Asymmetric Ion Mobility Spectrometry
KI: Kovats Index
KNN: K-Nearest Neighbors
LDA: Linear Discriminant Analysis
WDELM: Weighted Discriminative Extreme Learning Machine
GA: Genetic Algorithm
XGBoost: EXtreme Gradient Boosting
